# Cloning, expression and purification of functionally active human angiopoietin-like protein 2

**DOI:** 10.1186/2193-1801-3-337

**Published:** 2014-07-03

**Authors:** Nada Farhat, Aida M Mamarbachi, Eric Thorin, Bruce G Allen

**Affiliations:** Departments of Pharmacology, Montreal, QC H3T 1J4 Canada; Department of Surgery, Université de Montréal, Université de Montréal, 5000 Belanger St, Montréal, Québec H1T 1C8 Canada; Departments of Biochemistry, Montreal, QC H3T 1J4 Canada; Department of Medicine, Montreal Heart Institute, Université de Montréal, 5000 Belanger St, Montréal, Québec H1T 1C8 Canada; Montreal Heart Institute, Montréal, Québec H1T 1C8 Canada; Department of Pharmacology and Therapeutics, McGill University, Montréal, Québec H3G 1Y6 Canada; Pharsight Corporation Canada, 2000 Peel, Montreal, Québec H3A 2W5 Canada

**Keywords:** Angiopoietin-like protein 2, HEK 293 cells, Protein expression, Glycoprotein, Purification, Angiogenesis, Atherosclerosis

## Abstract

Angiopoietin-like protein 2 (Angptl2) is a secreted glycoprotein that has been implicated in angiogenesis, inflammation and atherosclerosis as well as enhancing the survival of human hematopoietic stem cells. Glycosylation of Angptl2 is required for biological activity and studies of angiopoietin-like protein 2 have been hindered by the lack of a source for the mature form of this protein. We describe a system that permits purification of the glycosylated form of human Angptl2 from conditioned media of stably transfected HEK 293 cells. To facilitate purification while retaining the integrity of Angptl2’s endogenous N-terminal secretion signal peptide, GST was fused downstream of the Angptl2 coding sequence. Secreted Angptl2-GST was purified using a one-step glutathione-affinity purification scheme. The purity and identity of the resulting protein were confirmed by SDS-PAGE, immunoblotting, and mass spectrometry. N-Glycosidase treatment reduced the apparent molecular mass of Angptl2-GST on SDS-PAGE, confirming its glycosylation state. Purified human Angptl2-GST stimulated both HUVEC migration and microtubule formation *in vitro*. The yield of Angptl2-GST obtained was in quantities suitable for multiple applications including functional *in vitro* and *in vivo* assays.

## Background

Angiopoietin like-2 (Angptl2) is a widely expressed, 57-kDa protein secreted into the circulation (Kim et al. 1999). Angptl2 is expressed in the heart, adipose tissue, stomach, small intestine, colon, ovary, uterus, spleen, striated muscle, and, at lower levels, in other tissues (Kim et al. 1999; Tabata et al. 2009) and Angptl2 is secreted by different cell types such as adipocytes (Tabata et al. 2009), endothelial cells (Farhat et al. 2013), macrophages (Tazume et al. 2012), keratinocytes (Ogata et al. 2012) and cancer cells (Endo et al. 2012). The primary structure of Angptl2 predicts an N-terminal coiled-coil domain, a C-terminal fibrinogen-like domain, as well as 2 sequential consensus sites for potential N-glycosylation and a hydrophobic region at the N-terminus typical of a secretory signal sequence (Kim et al. 1999). The coiled-coil domain appears to be sufficient for Angptl2 increased hematopoietic stem cell proliferation (Zhang et al. 2006; Broxmeyer et al. 2012). In contrast, alignment of the 7 angiopoietin-like proteins suggests that the fibrinogen-like domain is required for their angiogenic activities (Hato et al. 2008) and the fibrinogen-like domain of Angptl3 is sufficient to induce angiogenesis (Camenisch et al. 2002). Hence, Angptl2 may function as at least a bifunctional ligand in terms of its effects upon different target cell populations.

Although the Angptl2 receptor remains to be identified, a recent study identified the immune-inhibitory receptor human leukocyte immunoglobulin-like receptor B2 (LILRB2) and its mouse ortholog, paired immunoglobulin-like receptor (PIRB), as receptors for angiopoietin like-2, −5 and −7 in hematopoietic stem cells (Zheng et al. 2012). Recent findings report strong evidence that Angptl2 mediates chronic inflammation (Tabata et al. 2009; Endo et al. 2012; Ogata et al. 2012; Tazume et al. 2012; Farhat et al. 2013), making Angptl2 a potential therapeutic target, but there are currently no inhibitors or antagonists available to facilitate studies of the intracellular signaling pathways activated by Angptl2. Furthermore, although mammalian cell-expressed Angptl2 increased hematopoietic stem cell proliferation, bacterially expressed Angptl2 did not, suggesting mammalian-specific post-translational modification, likely glycosylation, of Angptl2 that contributes to, or is required for, one or more of its physiological functions (Zhang et al. 2006). Full length Angptl2 having mammalian cell-type glycosylation is not currently commercially available, which is an impediment to the further study of the physiological function and down stream signaling of Angptl2. Although some studies report the use of recombinant glycosylated Angptl2 protein, there are almost no methodological details concerning its preparation and purification (Zhang et al. 2006; Akhter et al. 2013; Farhat et al. 2013). Our objective was therefore to describe the cloning, expression, and purification of the mature glycosylated form of human Angptl2 as a GST fusion protein, Angptl2-GST. Purified, recombinant Angptl2-GST retains its expected pro-angiogenic and chemotactic effects on cultured HUVEC cells, indicating it is able to bind and activate its cognate receptor, suggesting that it is suitable for multiple applications including functional *in vitro* and *in vivo* assays.

## Results and Discussion

### Expression and purification of recombinant Angptl2-GST

**Figure 1:**
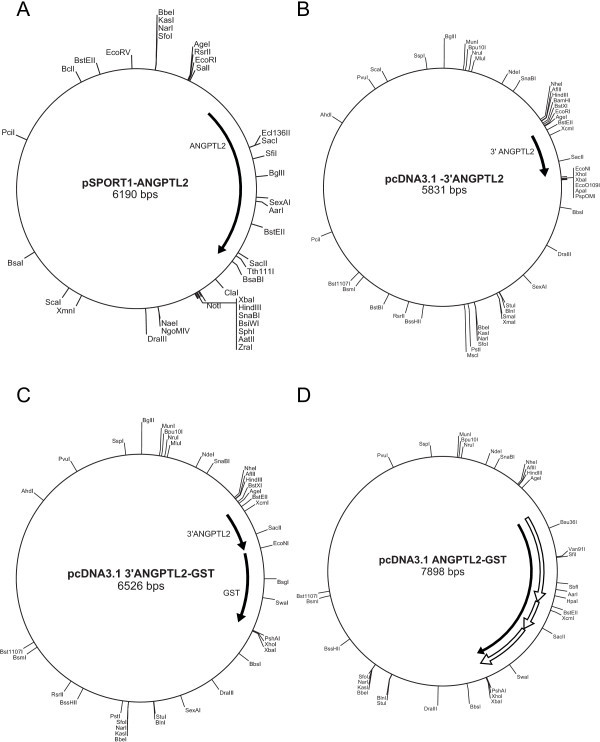
**Strategy for constructing human Angptl2-GST. A)** Map of the SPORT1-hAngptl2 vector obtained from OpenBioSystems. **B)** PCR amplification of a fragment corresponding to the 3’ region of Angptl2 without the stop codon and subcloning it into the pcDNA3.1 vector. **C)** Creating a fusion constructs containing the 3’ fragment of Angptl2 plus GST. **D)** Full-length Angptl2-GST construct (solid arrow) indicating the fragments used in its assembly (open arrows).

**Figure 2:**
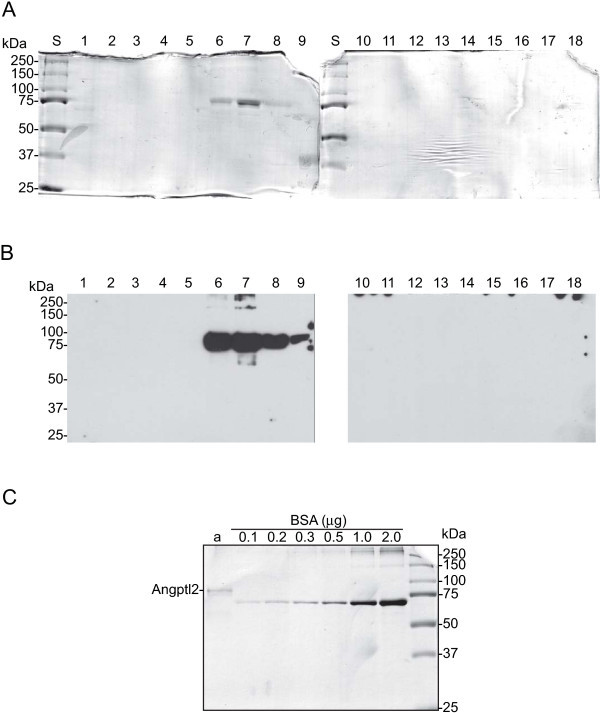
**Purification of recombinant human Angptl2-GST from conditioned media** ***.*** After loading the GSTrap column with 2–2.5 liters of conditioned media, the column was washed with TBSE and then eluted with 10 mM glutathione in TBSE. The fractions obtained during the elution of Angptl2-GST from the GSTrap column were resolved on SDS-PAGE to assess the specificity of the purification. **A)** Following SDS-PAGE, proteins were detected by staining with Coomassie Brilliant Blue. Lane 1 of each gel contains molecular mass markers. Lanes 2–10 contain the indicated fraction number (5 μl each). **B)** Immunoblotting of the GSTrap elution profile. The samples loaded were 0.1 μl of the same fractions in panel A, loaded on a separate set of 10% acrylamide gels and Immunoblotted as described in Methods. The membranes were probed using an antibody specific for human Angptl2 antibody. **C)** Quantification of purified Angptl2-GST. An aliquot (2 μl) of purified Angptl2-GST was resolved on SDS-PAGE (10% acrylamide) along with a standard curve of BSA (0.1, 0.2, 0.3, 0.5, 1 and 2 μg). The gels were stained with Coomassie Brilliant Blue R-250, digitized using a two-dimensional gel scanner, and band intensities for BSA and Angptl2-GST determined using Quantity One software and the quantity of Angptl2-GST determined from a linear regression analysis of a plot of band intensity versus micrograms of BSA loaded using GraphPad Prism software.

Angiopoietin-like protein 2 is a glycoprotein that is expressed in many tissues (Kim et al. 1999). Bacterially expressed Angptl2 is unable to stimulation expansion of hematopoietic stem cells, indicating that appropriate posttranslational modification is required for Angptl2 to be functional (Zhang et al. 2006). To this end, a line of stably transfected HEK 293 cells expressing human Angptl2 as a GST-fusion protein (Angptl2-GST) was created. Briefly, the full-length cDNA for human Angptl2 was obtained from OpenBioSystems in a pSPORT1 vector (clone ID LIFESEQ2268890; Figure 1A) and subcloned into pcDNA3.1 as described in METHODS and summarized in Figure 1. The resulting construct comprised the full-length Angptl2 coding sequence followed by GST (pcDNA3.1-Angptl2-GST; Figure 1D). pcDNA3.1-Angptl2-GST was transformed into E. coli DH5α competent cells, amplified, purified, and verified by sequencing.Following sequence validation, HEK 293 cells were transfected with pcDNA3.1-Angptl2-GST. Twenty-four h after transfection, the media was replaced with fresh DMEM supplemented with 1 mg/ml G418 and HEK 293 cells were cultured in the presence of G418 to select for stably transfected cells expressing Angptl2-GST. Once a stable line was obtained, their ability to express and secrete Angptl2-GST was assessed. Angptl2-GST was purified from conditioned media using glutathione affinity chromatography on 1 ml GSTrap FF columns (GE Healthcare; Figure 2). The elution of Angptl2-GST from glutathione Sepharose was assessed by separating an aliquot of each fraction on SDS-PAGE and visualizing the proteins using Coomassie Brilliant Blue R250 (Figure 2A). The identity of the protein eluting from glutathione Sepharose as Angptl2-GST was confirmed by immunoblotting using an Angptl2-specific antibody (Figure 2B). Purified recombinant Angptl2-GST migrated on SDS-PAGE with an observed molecular mass of ~90 kDa (Figure 2C), which corresponds with that predicted for Angptl2 (64-kDa) plus GST (28-kDa). The identity of this band as Angptl2-GST was further confirmed by tandem MS/MS.

Tandem MS/MS verification of expressed human angiopoietin-like protein 2. To confirm the expressed, purified protein was human angiopoietin-like protein 2, the 90-kDa band revealed by Coomassie staining was excised and submitted to the IRIC Proteomics Core facility for sequencing by LC-MS/MS (http://www.iric.ca/en/research/core-facilities/proteomics/). The unique peptides identified by MS are in bold in the sequence shown below. Similar results were obtained following analysis performed in 3 separate preparations of human Angptl2. In total, 12 unique peptides were identified, representing 88 out of a total of 493 amino acids and hence 17.8% sequence coverage.

1. mrplcvtcww lgllaamgav agqedgfegt eegsprefiy lnrykrages qdkctytfiv
2. pqqrvtgaic vnskepevll enrvhkqele llnnellkqk rqietlqqlv evdggivsev
3. kllrkesrnm **nsrvtql**ym**q llheii**rkrd n**alelsqlenr**ilnqtadml **qlask**ykdle
4. hkyqhlatl**a hnqsei**iaq**l eehcqrvpsa** rpvpqpppaa pprvyqppty nriinqi**stn**
5. **eiqsdq**nlkv lppplptmpt ltslpsstdk psgpwrdclq aledghdtss iylvkpentn
6. rlmqvwcdqr hdpggwtviq rrldgsvnff rnwetykqgf gnidgeywl**g leniywltnq**
7. **g**nykllvtm**e dwsgrk**vfae yasfrlepes eyyklrlgry hgnagdsftw hngkqfttld
8. rdhdvytgn**c ahyqkggw**wy nac**ahsnl**ng vwyrgghyrs ryqdgvywae frggsyslkk
9. vvmmirpnpn tfh

The yield of this expression system ranged from 25 to 100 μg of pure human Angptl2-GST per liter of conditioned media loaded onto the GSTrap columns. Digitizing the Coomassie-stained gels and assessing protein purity using Quantity One software (Bio-Rad Laboratories, Inc.) revealed the purity of Angptl2-GST to be > 95%. Each purification comprised 2–2.5 liters of conditioned media, which represented 6 weeks of cell culture. The number of flasks maintained in culture was adjusted so that the columns were loading continuously, via a 150 ml Superloop, at 0.2 ml/min. Higher flow rates were not sustainable over the long term, as the column bed compacted and back pressure increased beyond the limit of the GSTrap columns (0.3 MPa). Although the conditioned media was centrifuged prior to loading into the 150 ml Superloop, the columns were further protected by placing an in-line filter between the Superloop and the column. All buffers were filtered to 0.2 μm. Furthermore, the entire FPLC system was maintained at 5°C in a refrigerated chromatography cabinet. As Angptl2-GST has been shown to be pro-inflammatory, the endotoxin content was examined and found to be less than 1 endotoxin unit (EU) per microgram of purified Angptl2-GST. This value is comparable to full-length recombinant Angptl2 purified from E coli or truncated recombinant Angptl2 purified from human cell expression systems currently available from commercial sources.

### Purified Angptl2-GST is glycosylated

**Figure 3:**
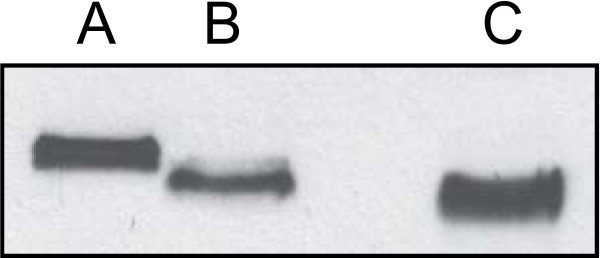
**Deglycosylation of Angptl2-GST.** Purified Angptl2-GST was incubated with in the absence (Lane **A**) or presence of PNGase F (Lane **B**) at 37°C for 5 h and then, along with commercially available human GST-Angptl2 (Novus Biologicals; Catalog number H00023452-P01; Lane **C**), resolved on SDS-PAGE (10% acrylamide), transferred to nitrocellulose (0.2 μm), and then visualized by immunoblot analysis using an Angptl2-specific antibody.

Endogenous Angptl2 is glycosylated and posttranslational modification of Angptl2 appears to be required for it to be functionally active with respect to regulating the activities of the target cell (Zhang et al. 2006). Kim and coworkers have previously shown that post translation modification increases the apparent molecular weight of Angptl2-GST on SDS-PAGE from 57-kDa to greater than 64-kDa (Kim et al. 1999). SDS-PAGE resolved purified Angptl2-GST into 3 bands (Figure 3), which likely correspond to different posttranslationally modified states of Angptl2. To confirm the glycosylation state of purified recombinant human Angptl2-GST, aliquots were incubated in the presence or absence of peptide N-glycosidase F (PNGase F), an amidase that cleaves between the GlcNAc and asparagine residues of N-linked glycoproteins, thus removing both high mannose and complex glycosylations. Following digestion of Angptl2-GST with PNGase F, only a single band of 57-kDa was observed (Figure 3), having an apparent molecular mass similar to commercially available, inactive, recombinant GST-Angptl2 (Novus Biologicals). Thus, like endogenous Angptl2, recombinant Angptl2-GST purified from media conditioned by stably transfected HEK 293 cells is also N-glycosylated.

### Functional assays of purified recombinant Angptl2-GST

**Figure 4:**
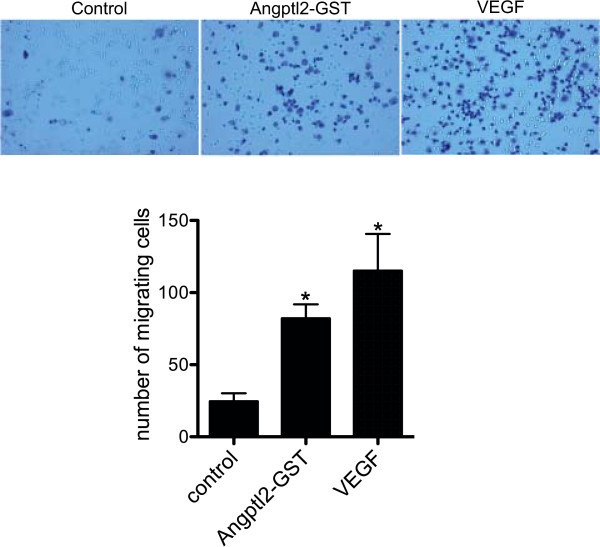
**Angptl2-GST promotes endothelial cell migration. Upper Panels)** Phase contrast images showing HUVECs that have migrated across the polycarbonate membranes (porosity 5 μm) separating the chambers in the transwells under the following conditions: culture media (Control), supplemented with 1 nM Angptl2-GST or 25 nM VEGF. **Lower Panel)** Histogram showing the number of cells having migrated across membrane. For each experiment, conditions were assayed in duplicate and 5 randomly chosen fields were quantified per condition. Data shown are mean ± SEM; n = 4; *, p < 0.05 versus control.

**Figure 5:**
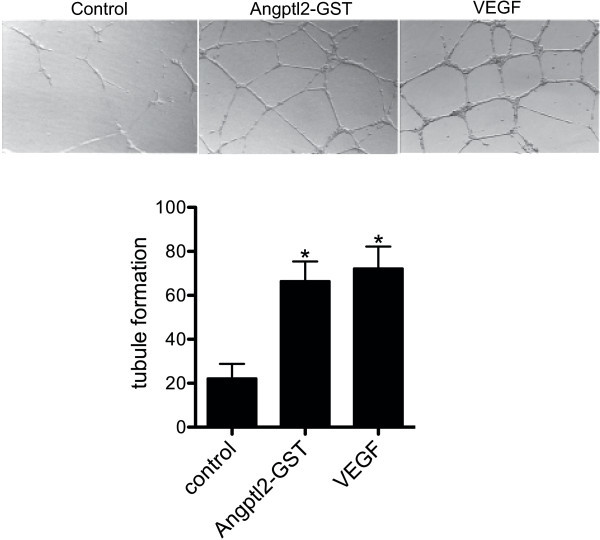
**Angptl2-GST promotes the formation of tubules in cultured HUVEC cells. Upper Panels)** The effect of Angptl2-GST on the ability of HUVECs to form a tubular network was assessed using an inverted microscope (400 ×). HUVECs were cultured on Matrigel-coated plates (on 6-well plates) for 24 h at 37°C and then stimulated, or not (control), for an additional 48 h with 1 nM Angptl2-GST or 25 nM de VEGF. **Lower Panel)** Histogram showing the number of adhesion points between microtubules. For each experiment, conditions were assayed in duplicate and 5 randomly chosen fields were quantified per condition. Data shown are mean ± SEM; n = 4; *, p < 0.05 versus control.

Several studies have shown that Angptl2 is pro-angiogenic: it stimulates the formation of new blood vessels in the Zebra fish (Kubota et al. 2005) and has been implicated in the maturation of blood vessel and the formation of arteries (Tabata et al. 2009). In addition, Angptl2 stimulates HUVECs to form tube-like structures (Kim et al. 1999). Studies have also shown that Angptl2 serves as a chemical attractant, stimulating the migration of monocytes/macrophages in culture (Tabata et al. 2009) and in the native endothelium (Farhat et al. 2013). To determine if purified recombinant Angptl2-GST is able to recapitulate the chemotactic and pro-angiogenic effects of native Angptl2, we assessed both of these properties *in vitro*. To examine the effects of Angptl2-GST on endothelial cell migration, HUVECs were plated in the upper chamber of transwell inserts and cultured in the presence of vehicle, Angptl2-GST, or VEGF in the lower chamber. Figure 4 shows Angptl2-GST increased the migration of HUVEC across the insert (from 25 ± 5 to 82 ± 10 cells/field, p < 0.05) to a similar extent as that observed with VEGF (from 25 ± 5 to 115 ± 26 cells/field, p < 0.05). Microtubule formation is an indicator of angiogenesis. Hence, to determine if Angptl2-GST is able to promote microtubule formation, HUVECs were seeded onto Matrigel-coated plates and stimulated with vehicle, Angptl2-GST, or VEGF for 48 h. Angptl2-GST increased microtubule formation (from 22 ± 7 to 67 ± 9 AU, p < 0.05) to a similar extent as did VEGF (from 22 ± 7 to 72 ± 10 AU, p < 0.05) (Figure 5). Taken together, these results confirm that Angptl2-GST is functional in that it promotes microtubule formation and chemotaxis as shown previously for the endogenous Angptl2. Thus the recombinant human Angptl2-GST obtained as described herein can be used to study the physiological and pathological roles of Angptl2, and we recently reported that Angptl2-GST is highly pro-inflammatory and induces atherogenesis in mice when injected *in vivo* (Farhat et al. 2013).

Studies characterizing the biological versus pathological function of a protein often include an examination of effects evoked by addition of the protein of interest. In the case of Angptl2 such studies are difficult due the lack of commercial availability of the protein. To date, commercial sources provide the full-length recombinant form of Angptl2. These sources use transgenic plant cells or E coli for the expression of Angptl2 as they provide an inexpensive means of expressing large amounts of the protein. However, these expression systems are unable to catalyze the pattern of glycosylation characteristic of eukaryotic glycoproteins (Ma et al. 2003). In order to express a functional recombinant form of Angptl2, it is essential to use a mammalian cell system. Due to the presence of an N-terminal signal sequence, the expressed Angptl2 is processed by the ER-Golgi system and secreted constitutively into the culture medium. By including a C-terminal GST moiety, we were able to employ a single-step protocol for the enrichment and purification of Angptl2-GST from conditioned media using GSTrap columns coupled with an FPLC system. The major drawback to such a system is the relatively low recovery of Angptl2, but this is circumvented by the fact that the concentration of Angptl2-GST needed to obtain a functional effect is in the order of nM. To the best of our knowledge, this is the first full description of the cloning, expression and purification of a mature glycosylated form of recombinant Angptl2.

## Conclusions

Angiopoietin-like protein 2 is a glycoprotein that is secreted by numerous cell types and has been shown to promote angiogenesis, inflammation and atherosclerosis as well as the survival of human hematopoietic stem cells. We have developed a protocol to express and purify human Angptl2-GST. A line of stably transfected HEK 293 cells was employed, as a mammalian cell system provides for the appropriate posttranslational modification of the recombinant protein. Subsequent analysis confirmed that the purified recombinant protein was glycosylated and retained the ability to promote endothelial cell migration and microtubule formation as reported previously for the endogenous Angptl2. Hence, our method of preparing purified recombinant human Angptl2-GST can be used to study the role of this protein both *in vivo* and *in vitro*.

## Methods

### Construction of a recombinant pcDNA-Angptl2-GST expression plasmid

#### Subcloning the 3’ of Angptl2 into pcDNA3.1

The full-length cDNA for human Angptl2 was obtained from Open Biosystems (pSPORT1-Angptl2; clone ID LIFESEQ2268890) (Figure 1A). A 3’ fragment of Angptl2, corresponding to nucleotides 1065–1479, was PCR amplified from pSPORT1-Angptl2 using primers that excluded the stop codon and added specific restriction cloning sites at the 3’ and 5’ ends. More specifically, the primers were designed as follows: 1) The ‘sense’ primer comprised sequence upstream of the BstEII site plus restriction sites specific for EcoRI and AgeI (Table 1); underlined; 2) the ‘antisense’ primer was composed of the 3’ sequence of Angptl2, excluding the stop codon, plus EcoNI (in phase with the Angptl2 coding sequence) and Xho1 sites (Table 1; underline). A fragment corresponding to the 3’ region of the Angptl2 coding sequence was amplified by PCR using Elongase and Elongase Enzyme Mix (Invitrogen) according to the manufacturer’s instructions. Briefly, 1 μl of plasmid DNA was combined with 1 μl of 10 mM dNTP, 1 μl each of sense and antisense primers (1 mM stock), 8 μl buffer A (5× stock comprising 300 mM Tris-SO_4_ (pH 9.1 at 25°C), 90 mM (NH_4_)_2_SO_4_, and 5 mM MgSO_4_], 2 μl buffer B [5× stock comprising 300 mM Tris-SO_4_ (pH 9.1 at 25°C), 90 mM (NH_4_)_2_SO_4_ and 10 mM MgSO_4_] and the final reaction volume adjusted to 50 μl with DNase-free H_2_O. Plasmid DNA was denatured at 94°C for 30 s and amplified for 30 cycles as follows: 30 s at 94°C (denaturation) followed by 30 s at 55°C and finally 45–60 sec at 68°C (elongation). The resulting 449 bp PCR product was resolved on a 2% agarose gel, the band excised, and the DNA extracted from the gel using a Gel Extraction Kit (Qiagen) (Figure 1B). To insert the 3’ Angptl2 cDNA fragment into the pcDNA3.1 neo (+) plasmid (Invitrogen), it was first necessary to modify the ends of the insert to be compatible with the vector. To accomplish this, vector and purified PCR product were digested with EcoRI. The EcoRI was then inactivated (70°C, 20 min) and a second digestion performed using XhoI. Between the first and second digestions, the EcoR1 buffer was removed by centrifugal filtration (Microcon YM-30 centrifugal filters, Millipore) and replaced with a buffer appropriate for EcoNI. The digested insert and vector were gel-purified and then ligated. The resulting pcDNA3.1-3’Angptl2 plasmid was transformed into competent DH5α cells, amplified, extracted and purified using MiniPrep kits. The presence and integrity of the insert was confirmed by digestion with EcoRI/EcoNI followed by agarose gel electrophoresis and sequencing.

**Table 1:** **Primers used for cloning human angiopoietin-like protein 2**

Primer name	Sequence
Angptl2’_S	
Angptl2’_AS	

The primers employed in the cloning of human angiopoietin-like protein 2 were designed to create 4 novel restriction sites. The nuleotides comprising each site are underlined and in italics with the endonuclease indentified immediately below.

#### Introducing GST cDNA

To preserve the functional integrity of the secretion signal peptide within the N-terminus of Angptl2, GST was inserted 3’ to Angptl2. The glutathione S transferase (GST) coding region was excised from the pGEX-6P-2 vector (Amersham) by digestion with XhoI (3’) followed by EcoNI (5’) and then purified on a 2% agarose gel. The pcDNA3.1-3’Angptl2 (nucleotides 1065–1479) plasmid was similarly digested with XhoI followed by EcoNI and the GST fragment was inserted in-frame 3’ to the Angptl2 coding region (Figure 1C). The product, pcDNA3.1-3’Angptl2-GST, was then transformed into competent bacteria, amplified, purified, and the integrity of the insert determined by sequencing as described above.

#### Assembly of full-length Angptl2

A fragment of Angptl2 comprising nucleotides −304 to +1098 was excised from the pSPORT1-Angptl2 vector (clone ID LIFESEQ2268890) by digestion with AgeI and BstEII (Figure 1D) and then inserted into the pcDNA3.1-3’Angptl2-GST plasmid, also digested with AgeI and BstEII, resulting in reconstitution of the full-length Angptl2 coding sequence. pcDNA3.1-Angptl2-GST was transformed into E. coli DH5α competent cells, amplified, purified, and verified by sequencing.

### Stable expression of recombinant human Angptl2-GST in HEK293 cells

As Angptl2 is a glycoprotein (Kim et al. 1999), a eukaryotic expression system was employed to insure that the expressed protein underwent appropriate posttranslational modification. Low passage number HEK 293 cells were obtained directly from Invitrogen and maintained in DMEM containing 10% de FBS and 1% penicillin/streptomycin. HEK 293 cells were transfected with pcDNA3.1-Angptl2-GST using Lipofectamine 2000 (Qiagen). Twenty-four h after transfection, the media was replaced with fresh DMEM supplemented with 1 mg/ml G418. Once a stable cell line was established, the ability of the cells to secrete intact Angptl2-GST into the culture medium was verified by immunoblotting (Figure 2). Cells were washed twice with PBS and then cultured in serum-free DMEM. Forty eight hours later the media was recovered, centrifuged for 30 min at 13,000 rpm and 4°C, and 1 ml of the supernatant concentrated 50-fold using centrifugal filtration units (Amicon Ultra-0.5 ml; 10 kDa nominal molecular weight limit). To minimize the potential for product loss due to non-specific protein binding, the filter unit was blocked using 10% FBS in DMEM (30 min, 5000 × g, 4°C). The concentrated, conditioned media was then separated on 12.5% acrylamide SDS-PAGE, transferred to nitrocellulose, blocked, and probed using a goat Angptl2-specific antibody (R&D) diluted 1:200 in TBST containing 5% skim milk (Carnation).

Upon confirmation of a stable cell line secreting full-length Angptl2-GST, cells were aliquoted, suspended in 10% DMSO: 90% DMEM supplemented with 10% FBS and stored under liquid nitrogen.

### Purification of Angptl2-GST

Stably transfected HEK 293 cells expressing Angptl2-GST were cultured in 175 cm^2^ flasks and the conditioned media harvested every 2–3 days, centrifuged for 2 h at 40,000 rpm and 4°C (Beckman Type 45 Ti rotor) to remove dead cells and debris, and applied to 1 ml GSTrap columns at 0.2 ml/min, using an FPLC maintained at 5°C. Columns were equilibrated with buffer comprising 50 mM Tris–HCl, 150 mM NaCl, 1 mM EDTA, pH 7.5 at 5°C (TBSE). For each preparation of Angptl2-GST, 2–2.5 liters of conditioned media was applied to the columns. The columns were then washed with 10 ml TBSE and eluted with TBSE containing 10 mM reduced glutathione (pH 8.0 at 4°C). Twenty fractions of 0.5 ml were collected. To determine the elution and purity of Angptl2-GST, aliquots of each fraction were subjected to both SDS-PAGE and immunoblotting. A 5-μl aliquot of each fraction was resolved on 10% acrylamide SDS-PAGE and the proteins revealed using Coomassie Brilliant Blue R250. In addition, 0.1 μl was resolved on 12.5% acrylamide SDS-PAGE, transferred to nitrocellulose, and revealed using Angptl2-specific antisera (Figure 2B). Once identified, the fractions containing Angptl2-GST were pooled, concentrated by centrifugal filtration (Amicon Ultra-0.5 ml; nominal molecular weight cut-off 10-kDa), and dialyzed against TBSE (4 buffer changes, 1000-fold buffer volume, 10-kDa NMWT cut-off Spectra/Por dialysis membranes) at 4°C. The final concentration of Angptl2-GST in each preparation was determined by separating both Angptl2-GST and a standard curve of BSA (0.1, 0.2, 0.3, 0.5, 1 and 2 μg; Figure 2C) on SDS-PAGE.

The gels were stained with Coomassie Brilliant Blue R-250, digitized using a two-dimensional gel scanner, and band intensities for BSA and Angptl2-GST determined using Quantity One software (Bio-Rad Laboratories). The mass of Angptl2-GST was determined by linear regression analysis of a plot of band intensity versus micrograms of protein (GraphPad Prism Version 4.00 for Mac).

### SDS-PAGE Analysis

Proteins were denatured by heating for 5 min at 80°C in Laemmli sample buffer (final concentrations: 50 mM Tris–HCl pH 6.8, 0.5 M β-mercaptoethanol, 2.5% SDS, 0.4 M sucrose, 1 mM EDTA and 0.05% bromophenol blue) and separated on 10% acrylamide SDS-PAGE mini-gels for 90 min at 100 V. To reveal proteins, gels were stained in 45% (v/v) denatured ethanol, 10% (v/v) acetic acid containing 0.1% (w/v) Coomassie brilliant blue R-250 and destained in 20% (v/v) denatured ethanol containing 10% (v/v) acetic acid. Bands were quantified using Quantity One software (Bio-Rad Laboratories).

### Immunoblot Analysis

Following SDS-PAGE, proteins were transferred to nitrocellulose membranes for 90 min at 100 V and 4°C in transfer buffer (25 mM Tris base, 192 mM glycine, 5% methanol). Membranes were blocked for 2 h at room temperature using 5% fat-free milk in 25 mM Tris–HCl pH 7.5, 150 mM NaCl, 0.05% (v/v) Tween 20 (TBST). To detect Angptl2 immunoreactivity, membranes were incubated for 3 h at room temperature with a goat anti-Angptl2 antibody (R&D Systems Catalog #AF2084) diluted 1:200 in blocking buffer. After washing 3 times (10 min each) in TBST, membranes were incubated for 2 h with horseradish peroxidase-conjugated donkey anti-goat secondary antibody (Jackson ImmunoResearch Laboratories) diluted 1:10,000 in blocking buffer. Finally, after 3 additional 10 min washes in TBST, immunoreactive bands were revealed by chemiluminescence (PerkinElmer) using BioMax BML film (Kodak).

### Mass spectrometry

Analysis of purified Angptl2-GST by mass spectrometry was performed by the Proteomics Core facility at the Institute for Research in Immunology and Cancer in Montreal, Qc, Canada (http://www.iric.ca/en/research/core-facilities/proteomics/).

### Endotoxin Quantitation

The assessment of endotoxin content of the purified Angptl2-GST preparations was determined by the Limulus Amebocyte Lysate (LAL) method using LAL Chromogenic Endotoxin Qunatitation Kits from Pierce according to the Manufacturer’s instructions.

### Endothelial cell migration assay

To verify the ability of purified recombinant human Angptl2-GST to function as a chemioattractant, we examined its ability to stimulate HUVEC migration in 48-well plates containing polycarbonate transwell inserts (Neuro Probe Inc., 5 μm pore size) as described previously (Bernatchez et al. 2003). HUVECs were suspended in serum-free media at 10^6^ cells/ml and 20 μl of this solution was plated in the upper chamber. The low chamber contained 50 μl serum-free media alone or serum-free media supplemented with either VEGF (25 nM) or Angptl2-GST (1 nM). Plates were placed in an incubator at 37°C for 1 h. The cells remaining on the upper surface of the transwell insert were removed and those cells having migrated through to the lower surface were fixed and stained using the Diff-Quick staining kit (Fisher Scientific). Inserts were then dried overnight at room temperature and mounted on microscope slides using a drop of oil. The number of stained cells having migrated across the insert membrane was quantified under the microscope (40 ×) in 5 separate fields per sample. Each condition was performed in duplicate.

### Microtubule Formation

Human umbilical vein endothelial cells (HUVECs) were plated at 2 × 10^5^ cells/well into 24- well plates precoated with Matrigel. After 24 h, the media was replaced with fresh media containing Angptl2 (1 nM) or VEGF (25 nM). Twenty-four h later, images were acquired using BTV Pro software and the number of microtubules was determined in 5 representative fields in each well.

## Abbreviations

Angptl2: Angiopoietin-like protein 2
HUVEC: Human umbilical vein endothelial cell.

## References

[CR1] Akhter S, Rahman MM, Lee HS, Kim HJ, Hong ST (2013). Dynamic roles of angiopoietin-like proteins 1, 2, 3, 4, 6 and 7 in the survival and enhancement of ex vivo expansion of bone-marrow hematopoietic stem cells. Protein Cell.

[CR2] Bernatchez PN, Tremblay F, Rollin S, Neagoe PE, Sirois MG (2003). Sphingosine 1-phosphate effect on endothelial cell PAF synthesis: role in cellular migration. J Cell Biochem.

[CR3] Broxmeyer HE, Srour EF, Cooper S, Wallace CT, Hangoc G, Youn BS (2012). Angiopoietin-like-2 and −3 act through their coiled-coil domains to enhance survival and replating capacity of human cord blood hematopoietic progenitors. Blood Cells Mol Dis.

[CR4] Camenisch G, Pisabarro MT, Sherman D, Kowalski J, Nagel M, Hass P, Xie MH, Gurney A, Bodary S, Liang XH, Clark K, Beresini M, Ferrara N, Gerber HP (2002). ANGPTL3 stimulates endothelial cell adhesion and migration via integrin alpha vbeta 3 and induces blood vessel formation in vivo. J Biol Chem.

[CR5] Endo M, Nakano M, Kadomatsu T, Fukuhara S, Kuroda H, Mikami S, Hato T, Aoi J, Horiguchi H, Miyata K, Odagiri H, Masuda T, Harada M, Horio H, Hishima T, Nomori H, Ito T, Yamamoto Y, Minami T, Okada S, Takahashi T, Mochizuki N, Iwase H, Oike Y (2012). Tumor cell-derived angiopoietin-like protein ANGPTL2 is a critical driver of metastasis. Cancer Res.

[CR6] Farhat N, Thorin-Trescases N, Mamarbachi M, Villeneuve L, Yu C, Martel C, Duquette N, Gayda M, Nigam A, Juneau M, Allen BG, Thorin E (2013). Angiopoietin-like 2 promotes atherogenesis in mice. J Am Heart Assoc.

[CR7] Hato T, Tabata M, Oike Y (2008). The role of angiopoietin-like proteins in angiogenesis and metabolism. Trends Cardiovasc Med.

[CR8] Kim I, Moon SO, Koh KN, Kim H, Uhm CS, Kwak HJ, Kim NG, Koh GY (1999). Molecular cloning, expression, and characterization of angiopoietin-related protein. angiopoietin-related protein induces endothelial cell sprouting. J Biol Chem.

[CR9] Kubota Y, Oike Y, Satoh S, Tabata Y, Niikura Y, Morisada T, Akao M, Urano T, Ito Y, Miyamoto T, Nagai N, Koh GY, Watanabe S, Suda T (2005). Cooperative interaction of Angiopoietin-like proteins 1 and 2 in zebrafish vascular development. Proc Natl Acad Sci U S A.

[CR10] Ma JK, Drake PM, Christou P (2003). The production of recombinant pharmaceutical proteins in plants. Nat Rev Genet.

[CR11] Ogata A, Endo M, Aoi J, Takahashi O, Kadomatsu T, Miyata K, Tian Z, Jinnin M, Fukushima S, Ihn H, Oike Y (2012). The role of angiopoietin-like protein 2 in pathogenesis of dermatomyositis. Biochem Biophys Res Commun.

[CR12] Tabata M, Kadomatsu T, Fukuhara S, Miyata K, Ito Y, Endo M, Urano T, Zhu HJ, Tsukano H, Tazume H, Kaikita K, Miyashita K, Iwawaki T, Shimabukuro M, Sakaguchi K, Ito T, Nakagata N, Yamada T, Katagiri H, Kasuga M, Ando Y, Ogawa H, Mochizuki N, Itoh H, Suda T, Oike Y (2009). Angiopoietin-like protein 2 promotes chronic adipose tissue inflammation and obesity-related systemic insulin resistance. Cell Metab.

[CR13] Tazume H, Miyata K, Tian Z, Endo M, Horiguchi H, Takahashi O, Horio E, Tsukano H, Kadomatsu T, Nakashima Y, Kunitomo R, Kaneko Y, Moriyama S, Sakaguchi H, Okamoto K, Hara M, Yoshinaga T, Yoshimura K, Aoki H, Araki K, Hao H, Kawasuji M, Oike Y (2012). Macrophage-derived angiopoietin-like protein 2 accelerates development of abdominal aortic aneurysm. Arterioscler Thromb Vasc Biol.

[CR14] Zhang CC, Kaba M, Ge G, Xie K, Tong W, Hug C, Lodish HF (2006). Angiopoietin-like proteins stimulate ex vivo expansion of hematopoietic stem cells. Nat Med.

[CR15] Zheng J, Umikawa M, Cui C, Li J, Chen X, Zhang C, Huynh H, Kang X, Silvany R, Wan X, Ye J, Canto AP, Chen SH, Wang HY, Ward ES, Zhang CC (2012). Inhibitory receptors bind ANGPTLs and support blood stem cells and leukaemia development. Nature.

